# Valorization of Olive Mill Wastewater by Membrane Processes to Recover Natural Antioxidant Compounds for Cosmeceutical and Nutraceutical Applications or Functional Foods

**DOI:** 10.3390/antiox7060072

**Published:** 2018-05-23

**Authors:** Alberto Alfano, Luisana Corsuto, Rosario Finamore, Maria Savarese, Filomena Ferrara, Salvatore Falco, Giuseppe Santabarbara, Mario De Rosa, Chiara Schiraldi

**Affiliations:** 1 Department of Experimental Medicine, Section of Biotechnology Medical Histology and Molecular Biology, University of Campania “Luigi Vanvitelli”, via L. De Crecchio No. 7, 80138 Napoli, Italy; luisanacorsuto@hotmail.it (L.C.); rosario.finamore@unicampania.it (R.F.); milenaferrara@hotmail.com (F.F.); giuseppesantabarbara@gmail.com (G.S.); mario.derosa@unicampania.it (M.D.R.); 2 Olio DANTE s.p.a., Zona Industriale, 82016 Montesarchio, Italy; mariasavarese@gmail.com (M.S.); salvatore.falco@oliodante.it (S.F.)

**Keywords:** olive mill wastewaters, polyphenols, natural antioxidant, functional food, ultrafiltration membranes, nano-filtration membranes, cell culture, scratch assays

## Abstract

Olive oil boasts numerous health benefits due to the high content of the monounsaturated fatty acid (MUFA) and functional bioactives including tocopherols, carotenoids, phospholipids, and polyphenolics with multiple biological activities. Polyphenolic components present antioxidant properties by scavenging free radicals and eliminating metabolic byproducts of metabolism. The objective of this research project was to recover the biologically active components rich in polyphenols, which include treatment of olive oil mills wastewater, and, at the same time, to remove the pollutant waste component resulting from the olive oil manufacturing processes. With specific focus on using technologies based on the application of ultra and nanofiltration membranes, the polyphenols fraction was extracted after an initial flocculation step. The nano-filtration permeate showed a reduction of about 95% of the organic load. The polyphenols recovery after two filtration steps was about 65% *w*/*v*. The nanofiltration retentate, dried using the spray dryer technique, was tested for cell viability after oxidative stress induction on human keratinocytes model in vitro and an improved cell reparation in the presence of this polyphenolic compound was demonstrated in scratch assays assisted through time lapse video-microscopy. The polyphenols recovered from these treatments may be suitable ingredients in cosmeceuticals and possibly nutraceutical preparations or functional foods.

## 1. Introduction

Olive oil is the principal fat source of the traditional Mediterranean diet and, due to its high content of polyphenols, has been associated with numerous beneficial-human health properties [1]. A Mediterranean diet rich in olive supplies around 10–20 mg of phenolic compounds per day [2].

Polyphenols are naturally occurring compounds found largely in fruits, vegetables, cereals, and beverages and are characterized by powerful antioxidant activities [3,4].

In fact, due to their chemical structure, polyphenols are able to scavenge a wide range of reactive oxygen species (ROS) such as hydroxyl radicals and superoxide radicals. These are highly reactive oxygen molecules that form cross linkages with the collagen molecules causing a loss of skin elasticity and tone, which initiates the aging process [5,6].

Moreover, excessive production of ROS or impaired detoxification of these aggressive molecules causes oxidative stress that hampers the normal wound repair process [7].

There is considerable interest in the use of natural antioxidants for preventing UV-induced skin photo damage. UV-induced skin photo damage is one of the main causes of skin alteration and premature skin aging [8]. Cosmetic formulations enriched by natural active principles (or ingredients) are promising alternatives to several synthetic ingredients in cosmetics existing in the market to treat premature aging that present common adverse reactions such as allergic contact dermatitis, irritant contact dermatitis, phototoxic reactions, and photo-allergic reactions [9]. In addition, the long-term consumption of diets rich in plant polyphenols offers some protection against the development of cancers, cardiovascular diseases, diabetes, osteoporosis, and neurodegenerative diseases [3,4].

Olive Mill Wastewaters (OMWs) deriving from constitution water and water used for both dilutions of olive paste and washing may become an important source for the extraction of natural antioxidants. Additionally, they are often found in polyphenols and are most abundantly found in the form of oleuropein, the derivatives of hydroxytyrosol, tyrosol, and a great variety of anthocyanins, catechins, and other compounds. The composition is strongly influenced by the variety of olives and seasonality. Hydroxytyrosol is easy to purify from a mixture of other biophenols. From a liter of OMWs, it’s possible to obtain 1.2 g of hydroxytyrosol and about 0.4 g of flavonoids [10].

Other biophenols present in OMWs are tyrosol, caffeic acid, and vanillic acid for which anti-oxidant, anti-atherogenic, and anti-inflammatory actions have been attributed [11].

In the medical-pharmaceutical field, it has been demonstrated that the antioxidant properties of oleuropein, tyrosol, and hydroxytyrosol have great potential. In relation to the onset of various degenerative diseases and the role antioxidants play in the control of these processes, the uncontrolled production of free radicals in various biological systems has been a topic of discussion for many decades especially for pathologists, biochemists, and nutritionists [12,13,14,15].

In this research project, a polyphenols-based formulation was obtained, which recovers the polyphenolic fraction from OMWs by optimizing integrated membrane processes. This formulation also aimed to obtain purified water to be recycled in the olive oil manufacturing and is generally reusable in other processes (irrigation, machinery washing, etc.).

Depending on the oil extraction process (pressing or centrifugation), the characteristics and quality of the starting olives, and on the practice adopted for their collection, the composition of OMWs changes greatly [16]. 

Membrane processes showed a number of advantages for purifying and concentrating bioactive phenolic compounds from OMWs. Specifically, they present a low energy requirement, separation efficiency, and ease of scaling up. For all these reasons, they received increasing scientific interest. Cassano and collaborators used microfiltration (MF), ultrafiltration (UF), nano-filtration (NF), and reverse osmosis (RO) processes to recover and purify polyphenols from OMWs [17,18,19].

Three different processes were studied in order to identify the most effective strategy in obtaining the highest product yield by reducing costs. Wastewater pilot plant treatment technology was developed by using a tangential filtration membrane process. The treatment consisted of initial processing of the wastewater to remove the solid component still present. Therefore, a subsequent treatment occurs by using tangential filtration on ultra and nano-filtration membranes in order to obtain concentrated phenols, purified water, and organic components that are useful for anaerobic digestion in the production of energy. In particular, for the nano-filtration phases, it is possible to obtain concentrates that may be used in food or cosmetic preparations. In fact, in these fractions, most of the phenolic components with an antioxidant activity were found. Some of the fractions obtained from the vegetation water treated with filtration processes (ultrafiltration and nanofiltration retentate) were subjected to drying by atomization with spray-drying technology. The technique transforms a feed liquid into a solid dry particulate through contact with hot gas, which results in the flash evaporation of the solvent. The nano-filtration concentrate residues were dried using the spray dryer technique and tested on human epidermal cells in vitro to evaluate values and their capability to contrast oxidative stress and to promote skin repair. In particular, in this research work, samples (nanofiltration retentates) were biologically characterized at first with a viability assay. The MTT test (3,(4,5-dimethylthiazol-2)2,5 difeniltetrazolium bromide) was performed after oxidative stress induction using human immortalized keratinocytes (HaCaT) as an epidermal cell model in vitro. The MTT test also allowed us to select the best concentration to be tested in order to evaluate the bio-revitalizing effect of these samples by performing a scratch assay on HaCaT monolayers and evaluating reparation rate through the time lapse video-microscopy.

Therefore, here we present an eco-sustainable process that focuses on the application of membrane treatments of the olive mill wastewaters. We also aim to recover the active molecules concentrated. The latter may then be assessed as high added value products for their potential anti-oxidant activity and beneficial effect on the wound healing in the in vitro model. The application of molecules (NF retentate) may sustain the wastewater treatment costs by improving the overall environmental impact in olive mill processing.

## 2. Methods

### 2.1. Materials

The OMWs used in this work and the celite used for filtering were provided by Industria Olearia Biagio Mataluni SRL (Benevento, Italy).

For the microfiltration process, BECO INTEGRA LAB 220 P with nitrocellulose filters with a porosity of 2.0 μm to 3.0 μm (BECOPAD P550) with filtering surface of 0.077 m^2^ (Fluxa Filtri, Milano, Italy) was used.

The centrifugation process was performed at 6000 rpm for 30 min with the Beckman centrifuge Avanti J20XP (Beckman, Milano, Italy).

### 2.2. Downstream Process

For the purification of the olive mill wastewaters, three different process strategies were identified before UF/NF processes. In the first experiment, the sample was micro-filtered on nitrocellulose filters with a porosity of 2.0 μm to 3.0 μm. In the second case, OMWs were centrifuged at 6000 rpm for 30 min and, in the last trial, filtration with adjuvants and microfiltration was used.

#### 2.2.1. Flocculation and Microfiltration

For the flocculation, three different concentrations of celite were used.

In particular, 5 L of olive mill wastewater were treated with 0.5%, 1%, and 2% *w*/*v* of celite. The adjuvants earth was dissolved directly in water and the solution was stirred for 1 h.

Then, the sample was subjected to a microfiltration process using the system BECO INTEGRA LAB 220 P.

The system has a diameter of 220 mm and was equipped with a reservoir of 1.8 L, inlet and outlet lines, and a pressure gauge (0–6 bar).

#### 2.2.2. Ultrafiltration and Nano Filtration

**Figure 1:**
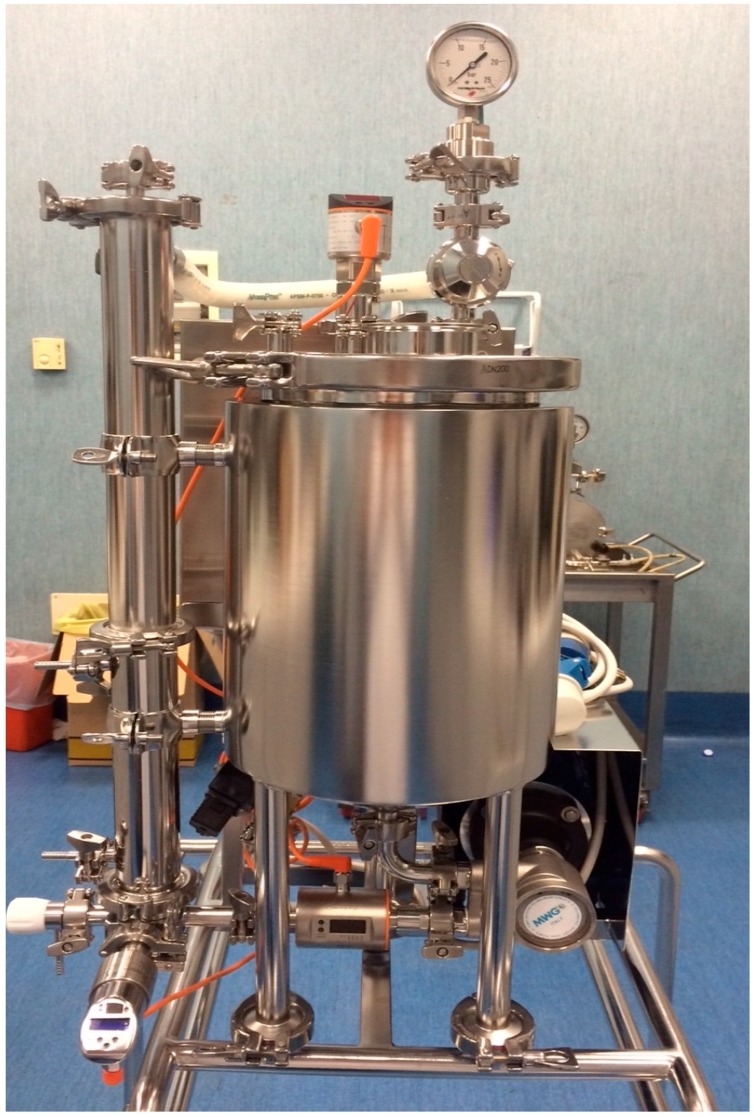
UF-NF (ultrafiltration-nano-filtration) system.

Ultrafiltration and nano filtration processes were carried out using a polyethersulfone spiral membrane from 100 KDa and 3–5 Da cut-off, respectively, with a total filtering area of 0.3 m^2^ (Fluxa Filtri, Milano, Italy). The system used for the membrane process was equipped with a 10 L volume tank, pressure gauges on the inlet, and retentate lines. Additionally, a thermostatic bath kept the temperature constant (see Figure 1).

### 2.3. Analytical Methods

#### 2.3.1. Methods for the Quantification of the Phenolic Fraction Extracted from the Olive Mill Wastewaters

The samples obtained were analyzed by using the HPLC system (model STH 575, Dionex) to determine the phenolic compounds from olive mill wastewater. The analyzed compounds include caffeic acid, hydroxytyrosol, tyrosol, oloeuropein, vanillic acid, coumaric acid, and syringic acid. Two buffers were used. Buffer A containing water and 0.1% *w*/*v* trifluoroacetic acid (TFA) and buffer B containing acetonitrile and 0.1% TFA *w*/*v*. The column used was C18 Zorbax dimethyl-octadecylsilane (ODS) 150 mm, id: 4.6 mm, particle size: 5 μm, temperature 25 °C and flux 1.1 mL/min, and volume injection was 20 μL. The gradient for HPLC analyses was: 0.0 min %B = 5.0, 2.0 min %B = 5.0, 12.0 min %B = 9.7, 42.0 min %B = 10.0, 45.0 min %B = 45.0, 49.0 min %B = 95.0, 52.0 min %B = 5.0, 55.0 min %B = 5.0.

On the other hand, in order to determine the carbohydrate component of wastewater mills, the samples were analyzed by high-performance anion exchange chromatography (HPAE-PAD, model ICS-3000, Dionex, CA, USA), according to the method of Marcellin et al. [20]. Both the standard of sucrose, fructose, glucose, and galactose were used to obtain the calibration curve and both treated samples on membranes of cut-off 3 kDa were analyzed, according to a method of separation using isocratic eluent NaOH 487 mM.

#### 2.3.2. Determination of Chemical Oxygen Demand (COD) Residual

The Chemical Oxygen Demand (COD) was measured according to method ISO 6060:1989 “Water quality—Determination of the chemical oxygen demand” [21].

### 2.4. In Vitro Biological Assays

#### 2.4.1. Cell Culture

For our purpose, the biological assays were run using HaCaT cells, which is a spontaneously transformed non-tumorigenic human keratinocyte cell line purchased by Istituto Zooprofilattico, Brescia, Italy. HaCaT were cultured, as previously reported in D’Agostino and collaborators [22]. Seeded multi-wells were incubated in humidified atmosphere (95% air, 5% CO_2_ *v*/*v*) at 37 °C. All cell materials were provided by Life Technology (Monza Italy). Collagen and Thiazolyl Blue Tetrazolium Bromide (MTT) were supplied from Sigma, Aldrich (Milan, Italy).

#### 2.4.2. Evaluation of the Cell Viability by MTT Assay after Stress Oxidative Induction

HaCaT (1.0 × 10^5^) cultured in a 24-well plate in Dulbecco’s Modified Eagle Medium (DMEM) medium were subjected to pretreatment with H_2_O_2_ 50 μM for 30 min and, successively, were treated with nanofiltration (NF) concentrate at 1 and 0.1 mg/mL. H_2_O_2_ 50 μM in medium represented the negative control. Growth medium (DMEM 10% fetal bovine serum FBS) was used as a positive control. The cell viability was checked by using the MTT assay, according to the method described by Mosmann et al. [23] after 24 h and 48 h of treatment.

#### 2.4.3. In Vitro Scratch Assay Using Time Lapse Video Microscopy (TLVM)

HaCaT cells were seeded at a density of about 1.8 × 10^5^ cells/well (4 × 10^4^ cells/cm^2^) in complete medium in 12-well tissue culture plates pre-coated with collagen, which was previously reported in D’Agostino et al., 2015 [22]. After two days, 2D monolayers were uniformly covering all the multiwall pavement. At this point, scratch wounds were manually obtained using a sterile pipette tip (Ø = 0.1 mm). Only uniformly sized wounds (e.g., 0.5–0.9 mm in width) were used for on line time lapse imaging and, therefore, were further analyzed. The scratched monolayers except for the control wells were incubated with 0.1 mg/mL of the polyphenols nano-concentrate to evaluate their potential ability in prompting wound reparation. The wound repair phenomenon was monitored for 72 h using TLVM (Okolab, Italy) to observe the migration and proliferation of HaCaT cells with and without the polyphenolic compound. The OKOLAB TLVM station, which was equipped with a stage incubator, allowed us to observe the scratch reparation occurring in different wells simultaneously and, at the end of the experiment, a specific software enabled us to build movies to compare the scratch reparations. In addition, quantitative analysis of wound healing was performed with OKO Vision software (Okolab, Naples, Italy) [22]. Experiments were run in triplicate and were repeated at least three times.

## 3. Results

### 3.1. Membrane Process Post Normal Filtration (First Strategy)

**Figure 2:**
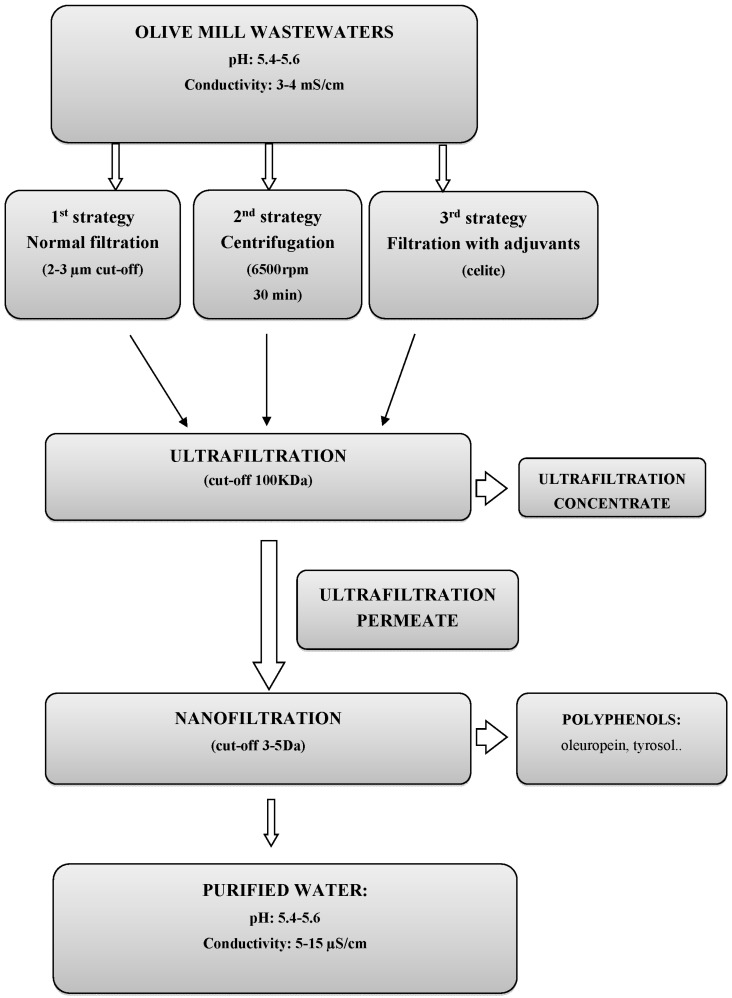
Block diagram of the process.

**Figure 3:**
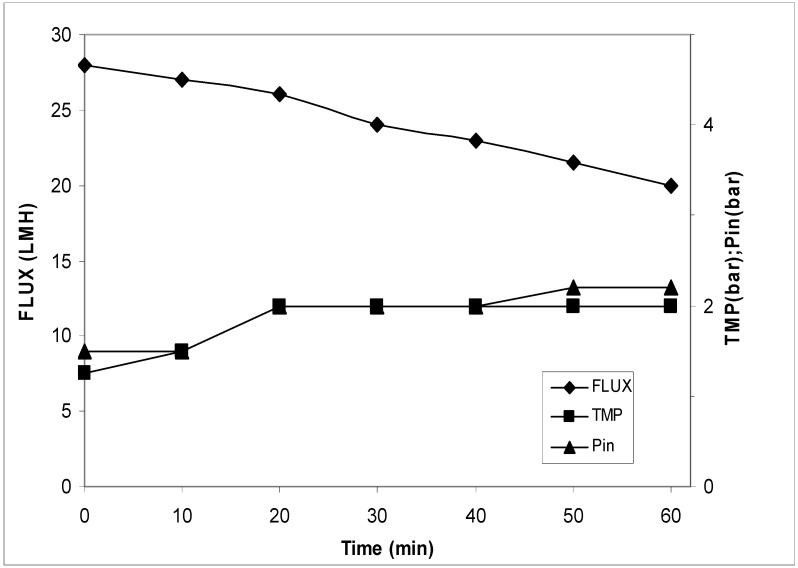
Transmembrane flux, corresponding transmembrane pressure (TMP), and inlet pressure during ultrafiltration treatment. The experiments were performed in triplicate.

**Figure 4:**
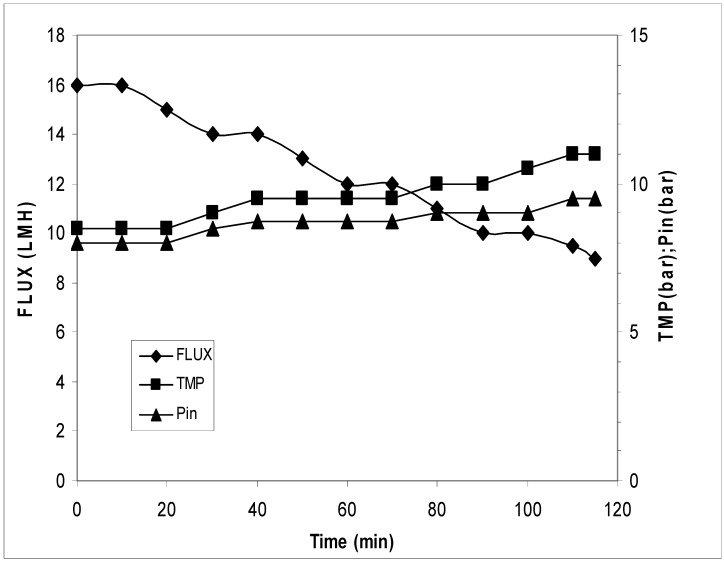
Transmembrane flux, corresponding TMP, and inlet pressure during nano filtration treatment. The experiments were performed in triplicate.

The block diagram of the process is reported in Figure 2. In these trials, the sample was filtered on 2 µm to 3 µm cut-off and then concentrated on the UF/NF membrane. The results of the UF process are reported in Figure 3. It can be seen that the flux had a reduction from 28.0 to 20.0 LMH (L/m^2^·h) and an increase of transmembrane pressure (TMP) from 1.2 bar to 2.0 bar. The concentration factor was around 20 fold. The results of the NF process are reported in Figure 4. The flux decreased from 16.0 LMH to 9.0 LMH and the TMP increased from 8.5 bar to 11.0 bar.

### 3.2. Membrane Process Post Centrifugation (Second Strategy)

**Figure 5:**
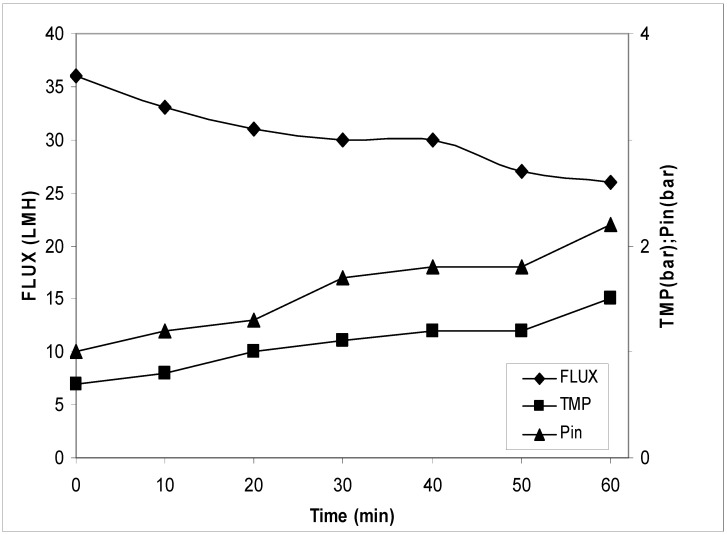
Transmembrane flux, corresponding TMP, and inlet pressure during the ultrafiltration treatment. The experiments were performed in triplicate.

**Figure 6:**
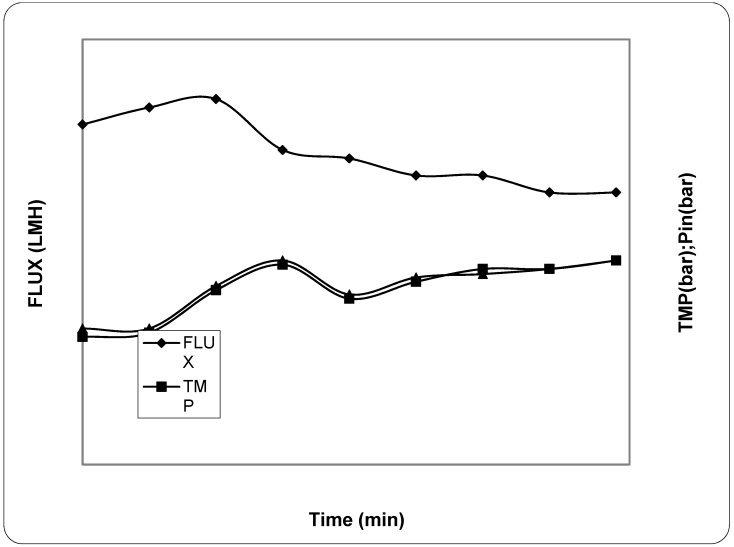
Transmembrane flux, corresponding TMP, and inlet pressure during the nano filtration treatment. The experiments were performed in triplicate.

In order to determine the weight of the pellet and evaluate whether the samples needed to re-centrifuge, tests were carried out on repeated centrifugation, 6000× *g*, for 30 min on the initial sample (see Table 1). The results obtained during the ultrafiltration process after centrifugation are reported in Figure 5. The flux had a reduction from 36.0 LMH to 26.0 LMH and an increase of transmembrane pressure (TMP) from 0.7 bar to 1.5 bar. The concentration factor was around 23.5 fold. The permeation obtained from the ultrafiltration treatment was subjected to the nano-filtration process. The results of this process are reported in Figure 6. The flux decreased from 20.0 LMH to 16.0 LMH and the TMP increased from 7.5 bar to 12.0 bar.

### 3.3. Membrane Process Post Treatment with Adjuvant (Third Strategy)

**Figure 7:**
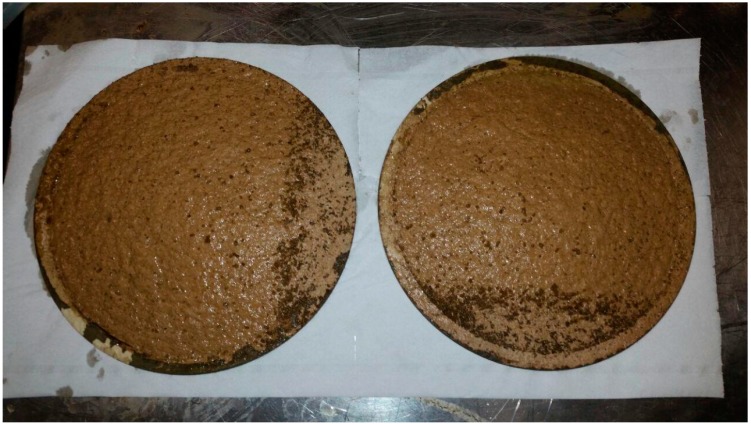
Filters BECOPAD 550.

**Figure 8:**
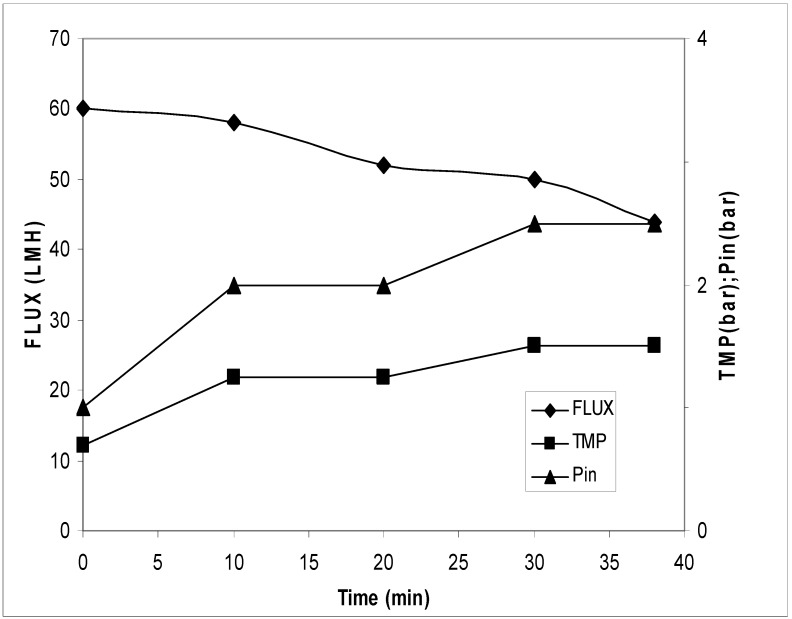
Transmembrane flux, corresponding TMP, and inlet pressure during the ultrafiltration treatment. The experiments were performed in triplicate.

**Figure 9:**
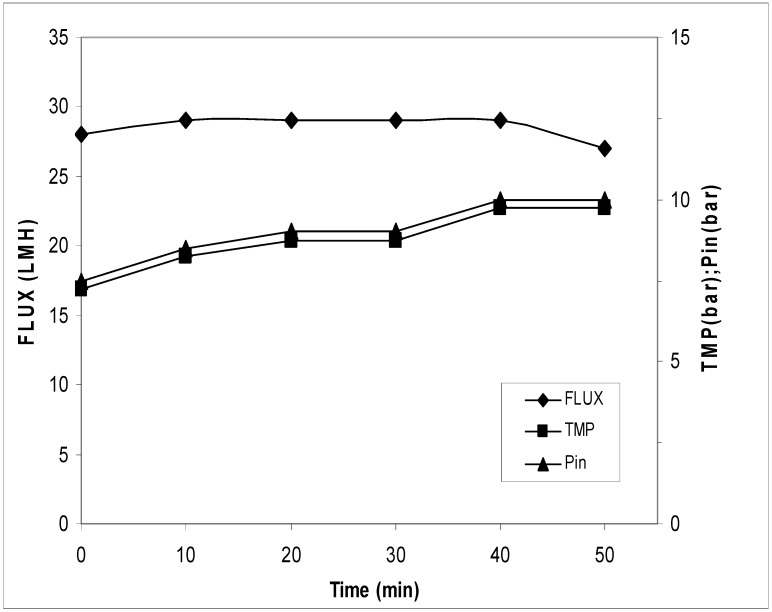
Transmembrane flux, corresponding TMP, and inlet pressure during the nano filtration treatment. The experiments were performed in triplicate.

The adjuvant was dissolved directly in the OMWs and the solution was stirred for 1 h and filtered through filters Beco 550. Figure 7 shows the filters after the treatment. After the filtration on the Beco disc, the sample was first ultra-filtrated and then nano filtrated. The results of the UF/NF processes were reported in Figure 8 and Figure 9. In the ultrafiltration process, the flux had a reduction from 60.0 LMH to 44.5 LMH and an increase of the transmembrane pressure (TMP) from 0.7 bar to 1.5 bar. The concentration factor was around 28 fold. In the nano filtration process, the flux was almost constant while the TMP increased from 7.2 bar to 9.7 bar.

### 3.4. HPLC Analysis

For the three different processes, the amount of polyphenols was evaluated by HPLC. Analyses were carried out in order to determine the amount of polyphenols present in the various steps of the process. The results obtained are reported in Table 2. In particular, 250 mg, 250 mg, and 430 mg of total polyphenols was content in the nano filtration retention of the first, the second, and the third strategy, respectively.

### 3.5. COD Analysis

Clear and transparent in appearance, the fraction typically shows a load of organic matter considerably lower than the initial vegetation water and can be recovered and reused in other processes within a company.

**Figure 10:**
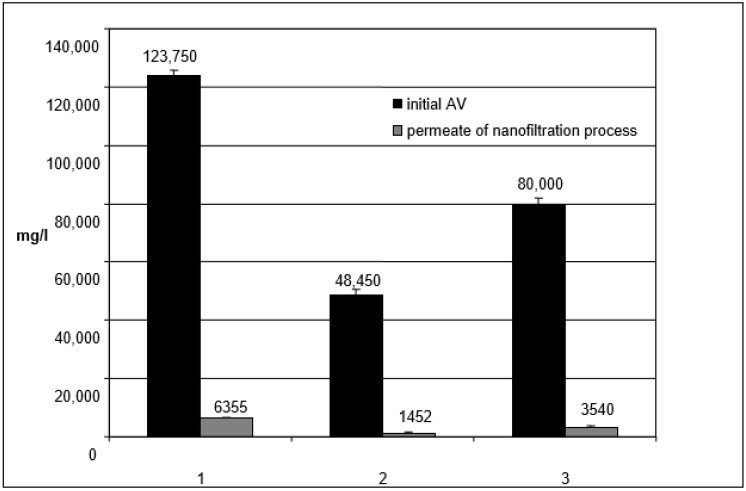
Chemical Oxygen Demand (COD) analyses in the initial wastewater and in the permeate of the nano filtration process. AV: vegetation water or wastewater.

To evaluate the effectiveness of the tangential filtration process, analysis of the COD in the initial wastewater (initial AV vegetation water or wastewater), and the permeate of the nano filtration process was performed. The data of three tests of treatment is shown in the graph of Figure 10. The results showed a reduction of 95.0% to 97.0% *w*/*v* of the organic load in all the strategies.

### 3.6. MTT Test

**Figure 11:**
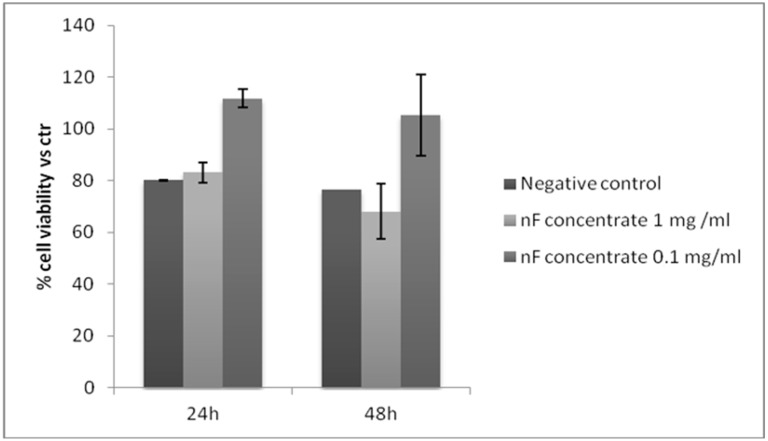
MTT assay for each concentration of the NF concentrate after 24 h and 48 h of treatment.

Following oxidative stress induction and treatment with the nano filtration sample derived from the third strategy at different concentration (1 mg/mL and 0.1 mg/mL), cell viability was determined by the MTT test after 24 h and 48 h of treatment. The results, which are reported in Figure 11, demonstrated that adding 1 mg/mL at 24 h did not protect from the oxidative stress and, in addition, at 48 h, cell death was evidenced similarly to the negative control. Cell viability at the lower concentration was >70% compared to the positive control after 24 h and 48 h. Therefore, according to ISO 10993-5 [24], the samples were not cytotoxic and even promoted cell proliferation (cell viability respect to control (CTR) > 100%).

### 3.7. Wound Healing Assay

**Figure 12:**
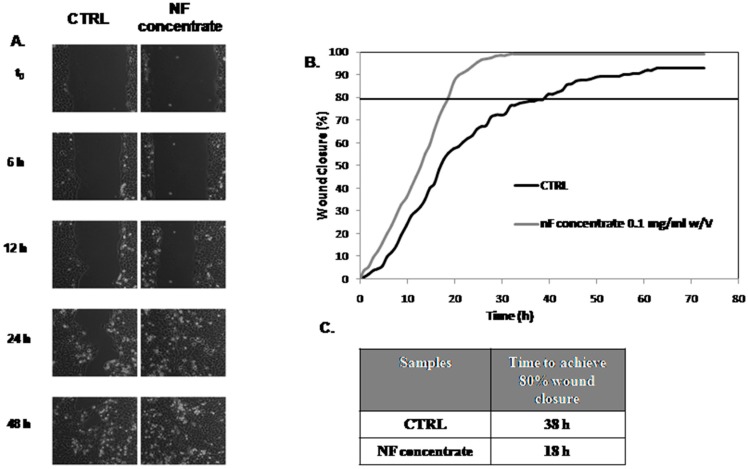
(**A**) Representative panel of comparison of wound repair with and without NF concentrate 0.1 mg/mL *w*/*v* at different times. (**B**) Repair represented by wound area reduction with time [as (Area t_0_ − Area t/Area t_0_) × 100] for the control and in presence of 100 for the control and in presence of NF concentrate. The curves are averages of three independent experiments with standard deviation within 5% of the value. (**C**) Average time to achieve 80% wound repair in the presence of NF concentrate 1 mg/mL compared to the control.

The biological effect of the 0.1 mg/mL NF concentrate, derived from the 3rd strategy, was also investigated on the wound healing process in HaCaT cell monolayers. Experiments were carried out using a stage incubator and time lapse video microscopy are shown in Figure 12. Polyphenols concentrate achieved 80% wound closure at 18 h that was about half the time needed for the control (standard growth medium). Overall, NF concentrate enhanced the scratch repair, which led to a complete re-epithelialization 24 h. This is shown in Figure 12, both in the comparative picture panel and in wound closure curves.

## 4. Discussion

The first target in this experimental project was to enhance wastewater mills treatment that, given their particular composition, has a significant environmental impact and is not conducive to agricultural land irrigation because of the risk of desertification and pollution of aquifers. The presence of polyphenols may be value added products and has to be addressed as a potential obstacle in the purification process (chemical nature).

In the Mediterranean area, the production of OMWs exceeds 15,000 cubic meters per year within a period of three to four months (November–February), which created a huge environmental problem. The biggest olive oil-producing country is Spain and Italy [19]. It is, therefore, of vital importance to propose a valid alternative to the land use of OMWs. In the Campania Region, approximately 500 mills annually produce roughly 140,000 cubic meters of vegetable water with a high pollution load due mainly to the high content of organic compounds. As a matter of fact, data in the referred literature illustrates that 1 m^3^ of wastewater mills has a pollution load equal to that produced by civil waste from 100,000 inhabitants. These numbers are important for understanding the potential environmental impact for the management of wastewater mills.

The choice of the membrane processes aims at reducing the pollutant load, to recover and utilize by-products, and to reuse water (circulating plant water). Overall, this approach will significantly reduce environmental impact.

In the retentate of the ultrafiltration step, an organic fraction impoverished of phenolic content but rich of substances is possibly usable for the production of biogas, are recovered. The UF permeate is loaded to NF membranes by obtaining a concentrated solution rich in the phenolic components, which is widely applicable.

In addition, there has been much interest in the potential health benefits of dietary plant polyphenols as antioxidants [25]. Therefore, the significance of these compounds are potential active ingredients in cosmeceutical, nutraceuticals, and functional foods.

Comparing the results of the different treatments exploited in the framework of this research project, we found that the strategy involves the use of adjuvants to remove undesired solid components, which is the one that leads to the greatest recovery of polyphenols.

Three different process strategies were identified in order to optimize the process by obtaining a high recovery of polyphenols at a low cost.

The best one exploited the flocculation adjuvants by obtaining a reduction of the organic content of about 30% to 35%. The supernatants obtained were subjected to UF and NF membrane processes in order to obtain a nano filtration permeate with low COD and a concentrate of nano filtration with a high content of polyphenols.

In the ultrafiltration process, the sample treated with this method (Figure 2, third strategy) showed a reduction of the flow from 60.0 LMH to 34.4 LMH with an increase of the transmembrane pressure (TMP) from 0.7 bar to 1.5 bar (see Figure 8). However, in the nano filtration process, an increase of transmembrane pressure from 7.2 bar to 9.7 bar permitted a constant flow of about 28.0 LMH (see Figure 9). As expected, higher fluxes were found in UF/NF processes when the wastewaters were previously treated with adjuvant. Fluxes recorded in this experimental work were slightly higher than those reported in the literature [18]. Compared to the other two strategies, the use of adjuvants besides improving yield reduced the processing time. In addition, the third strategy permitted to shorten the UF process time of about 30% with respect to the other two strategies. It can be argued that reducing process time likely led to lower energy and labor costs with economic improvement. HPLC analyses used for the quantification of the polyphenols show that, in the first treatment, recovery in the UF step was about 84% *w*/*v* and final recovery was 38% while, in the second treatment, recovery in the UF step was 80% and final recovery was 40%. In the third treatment, recovery on the UF step was 85% and final recovery was 64.7%, which was much better (40% increase) than the first two procedures (see Table 2).

Additionally, the process of microfiltration is simpler and cheaper when compared to a centrifugation process especially at an industrial scale. Furthermore, the celite used as adjuvant has a low cost and, therefore, minimal impact on the overall economy of the process.

The recovery of polyphenols helps to obtain added value from the waste treatment, which partially compensates the cost of the process.

Furthermore, the permeate of the nano filtration showed COD values ranging from 1500–6000 mg/kg with a reduction of about 95% of the organic load when compared to the initial wastewater (see Figure 10). The use of filtering lands/earths and membrane processes has allowed us to obtain a 30% higher COD removal when compared to processes that use chemical agents [26,27] and a reduction of COD similar to the processes using nano filtration and reverse osmosis in series [28].

This allows for water irrigation in fields and the use of the same water for the washing of manufacturing equipment.

NF concentrate promoted cell growth and exhibited protective action against oxidative stress but in a dose-dependent manner, which is found in MTT tests (see Figure 11 and Figure 12). In particular, the highest concentration assayed induced a decrease of cell viability when compared to the negative control.

Therefore, considering the best performance on cell viability and preservation of cell morphology in the presence of the 0.1 mg/mL NF concentrate at 24 h and 48 h, we investigated the bio-revitalizing effect of the polyphenolic sample by performing a scratch test in vitro on the keratinocytes cell line. The empirical and qualitative evaluation of wound repair was corroborated by quantitative evaluation of the TLVM device and software, which demonstrated the ability of polyphenolic concentrate to hasten the repair process with respect to the control. Therefore, polyphenolic fraction, recovered during the nano filtration process, proved to reduce oxidative stress and promote dermal regenerative bio-processes. These results suggest potential application in nutraceutical or cosmeceutical formulations.

## 5. Conclusions

The exploitation of UF and NF membranes technology, coupled with adjuvant based filtration, has allowed us to achieve the following environmental benefits: (i) recovery of water, which can be re-used in the productive cycle and contribute to significant saving of water resources, (ii) production of natural antioxidant extracts from an effluent, which, otherwise, would become waste. Our results may suggest an alternative procedure for OMW land-pouring, which, if carried out incorrectly, can lead to contamination of water courses or groundwater and negative effects for agriculture.

The membranes used proved to be washable and reusable, which led to a considerable reduction in costs and a decrease in environmental impact compared to biological and oxidation processes or treatments that involve the use of metals. Moreover, recovered polyphenols may be suitable active ingredients for natural cosmeceutical preparations or food additives.

## Figures and Tables

**Table 1:** Pellet weight post centrifugation.

Centrifugation	Pellet Weight (g/L)	% Weight Removed
I	0.471	71
II	0.096	14.5
III	0.073	11
IV	0.019	2.8

**Table 2:** HPLC analysis.

Treatment	To (mg)	UF_ret_ (mg)	UF_perm_ (mg)	Polyphenols Recovery UF Step (%)	NF_ret_ (mg)	NF_perm_ (mg)	Final Recovery (%)
1°	784 ± 15	125 ± 15	658 ± 15	84	250 ± 10	400 ± 12	38
2°	768 ± 15	153 ± 15	614 ± 10	80	250 ± 10	368 ± 15	40
3°	770 ± 15	115 ± 10	664 ± 15	85	430 ± 10	235 ± 10	64.7

UF: ultrafiltration; NF: nano-filtration.

## Abbreviations

OMWs	olive mill wastewaters
TSS	total suspended solid concentration
COD	chemical oxygen demand
BOD	biological oxygen demand
MWCO	molecular weight cut-off
MF	microfiltration
UF	ultrafiltration
NF	nano filtration
RO	reverse osmosis
HPLC	high performance liquid chromatography
LMH	flux normalized on surface area
TMP	transmembrane pressure
P_in_	inlet pressure
SE	standard error
HaCaT	human immortalized keratinocytes
